# Open questions on the physical properties of aerosols

**DOI:** 10.1038/s42004-020-00342-9

**Published:** 2020-08-07

**Authors:** Bryan R. Bzdek, Jonathan P. Reid, Michael I. Cotterell

**Affiliations:** School of Chemistry, University of Bristol, Bristol, BS8 1TS UK

**Keywords:** Atmospheric chemistry, Physical chemistry

## Abstract

Aerosols are highly dynamic, non-equilibrium systems exhibiting unique microphysical properties relative to bulk systems. Here the authors discuss the roles aerosols play in (bio)chemical transformations and identify open questions in aerosol-mediated reaction rate accelerations, aerosol optical properties, and microorganism survival.

Aerosols, which are solid particles or liquid droplets suspended in the gas phase, are central to a wide range of disciplines spanning climate science, energy and combustion, nanomaterials synthesis, drug delivery to the lungs, disease transmission, and consumer and agricultural products. Aerosols are highly dynamic, non-equilibrium systems, and their significance to these disciplines ultimately arises from unique qualities that derive from their microphysical properties. Aerosols have surface area-to-volume ratios orders of magnitude larger than those of macroscopic solutions, as highlighted in Fig. 1. Consequently, the importance of interfacial composition and reactivity is magnified in aerosols. Moreover, aerosols can access solute concentrations that are significantly higher than the saturation limit for macroscopic solutions. As an aerosol droplet dries, no heterogeneous nuclei or surfaces exist onto which crystallisation can occur. Consequently, extremely high solute supersaturations and ionic strengths are possible in aerosol droplets, potentially enabling unique chemical reactions. Figure 2 shows that an aqueous sodium nitrate droplet reaches a concentration equivalent to that of a saturated solution (~10 molal, i.e., moles of solute per kg solvent) near 75% relative humidity (RH) or, equivalently, a water activity of 0.75. At even lower RH values, instead of crystallising, the droplet remains liquid and theoretically reaches concentrations >10^3^ molal. Because of the chemical complexity and high solute concentrations accessible, aerosols can exhibit unique phase behaviour spanning non-viscous to ultraviscous or glassy phases as well as surprising phase separation behaviour and morphologies^1^. Aerosol morphology and composition are also highly size dependent. Additionally, predicting the consequences of light scattering or absorption by a particle depends on the microphysical model chosen to represent particle size, shape, and internal structure.

**Fig. 1:**
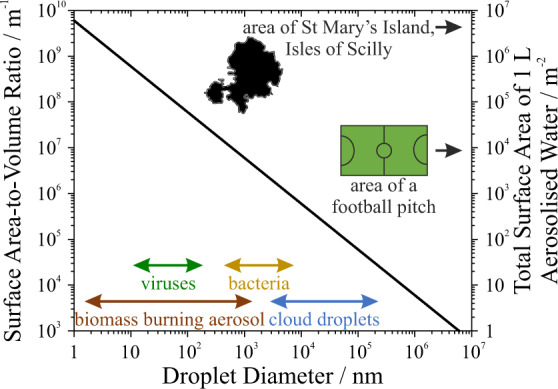
Aerosol surface area-to-volume ratios. Aerosols exhibit surface area-to-volume ratios that are orders of magnitude larger than those of macroscopic systems. The secondary *y*-axis illustrates how the total surface area of 1 L of liquid water changes as the liquid is aerosolised to a monodisperse distribution of a specific size.

**Fig. 2:**
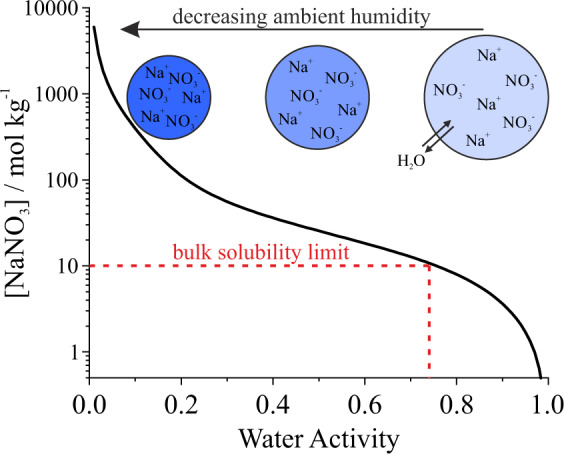
Supersaturation in aerosol droplets. Sodium nitrate is an example of a compound that often will not crystallise in the aerosol phase, instead reaching highly supersaturated solute states that are well beyond those accessible in macroscopic systems. The relationship between solute molality and water activity was calculated using the Aerosol Inorganics Model (http://www.aim.env.uea.ac.uk/aim/aim.php). Water activity = Relative humidity/100%.

The study of aerosols is challenging because these systems are highly dynamic and polydisperse, exhibit non-ideal properties, and contain very little mass. Approaches are still being developed to identify and quantify their unique chemical and physical attributes. In this comment, we highlight three areas where aerosol microphysics may be key to driving chemical or biochemical transformations: reaction rate enhancements in compartmentalised systems, transformations of light absorbing aerosol, and the viability of airborne pathogens.

## Aerosol microphysics drives enhancements in reaction rates

An increasingly large body of evidence concludes that some reactions proceed at much faster rates in aerosol droplets than in macroscopic solutions, with estimated rate enhancements sometimes exceeding 10^5^ ^2,3^. The origin of these reaction rate enhancements is poorly understood, but their observation has major implications in research areas spanning organic synthesis (enhanced yields, reduced waste, and straightforward scale-up), atmospheric composition and reactivity (pollutant production and transport), and the origin of life (abiotic production of complex, biologically significant molecules).

Reaction rate acceleration may be driven by multiple unique aerosol properties. For example, confinement effects may modify reaction thermodynamics such that reactions unfavourable in macroscopic systems become spontaneously favourable^4^. The high surface area-to-volume ratios of aerosol droplets can alter equilibrium surface composition relative to the bulk solution that produced the droplet^5^. Similarly, pH can vary spatially within a droplet and may differ from that in the bulk^6^. Solvent evaporation from the droplet drives large increases in solute concentrations and ionic strength (Fig. 2), resulting in significantly faster reaction rates relative to those measured in the bulk^7^. As droplet size decreases, diffusion timescales may become shorter, facilitating increases in reaction rates. Understanding the reasons for reaction rate acceleration in aerosol droplets requires determination of the relative importance of these often competing parameters.

A major limitation of many experimental investigations in this area is that droplet formation and chemical analysis are tightly coupled: in many studies, highly charged droplets are produced by electrospray ionisation and subsequently analysed by mass spectrometry within <<1 s^2^. Open questions in this area include: Are some aerosol microphysical properties more important than others in governing the magnitude of reaction acceleration? Do droplets that are generated, reacted, and analysed within milliseconds undergo reaction at equilibrium, or do composition gradients arise due to rapid evaporation or limited diffusion timescales? To answer these questions, theoretical treatments of compartmentalised reactions are required, as are the development of experimental approaches capable of decoupling the complex roles of charge, concentration (or evaporation), and the competition between surface and bulk reactivity.

## Aerosol microphysics drives transformations of aerosol optical properties

The representation of aerosols is one of the largest uncertainties in climate models. This uncertainty arises partly from knowledge gaps concerning the relative fraction of sunlight or infra-red terrestrial radiation that aerosols scatter and absorb, thereby affecting the radiative impacts aerosols exert via atmospheric cooling or heating. Inherently, aerosol optical properties are governed by the particle microphysical parameters including size, composition, and structure.

By way of example, biomass burning aerosol demonstrates particularly complex physicochemical interactions between directly emitted soot (amorphous carbon) particles and semi-volatile gases that rapidly condense to form organic “coatings”. The partitioning and chemical composition of semi-volatiles onto aerosols evolves over typical aerosol lifetimes of several days. The emerging picture, from field and laboratory measurements in combination with electromagnetic interaction models, shows condensed organics initially filling the voids between agglomerated carbon spherules of freshly emitted soot particles, before forming ever thicker surface films. Changes in surface tension during the partitioning of semi-volatiles cause the fractal soot agglomerates to collapse; the collapsed agglomerate provides a reduced particle surface area for light interactions thereby reducing the light absorption cross section^8^. Thick organic coatings in aged particles enhance light absorption by acting as effective lenses that focus light onto the strongly absorbing soot “core”. Moreover, the intrinsic optical properties of organic coatings undergo significant evolution. Organic chromophores that absorb in the short-visible spectrum are referred to as brown carbon (BrC). Recent field studies show that, while the organic mass fraction of a particle generally increases with age, the BrC chromophores are significantly depleted with increasing lifetime through photobleaching, hydrolysis, oxidation, or other physiochemical pathways^9,10^.

Chemical reactions of inorganic aerosol components (e.g., ammonium and nitrate species) with trace atmospheric gases also form BrC. The uptake and subsequent reactions of α-dicarbonyl gases with ammonium and amino containing aerosols has seen recent interest. Indeed, these reactions proceed on timescales of several days in bulk solution but are shown to occur within seconds in drying aerosols^11^. This observation highlights the need to understand fundamental reaction processes and the role of drying, particle size, electrostatic fields, and surface-mediated chemistry in resolving the evolution of BrC of direct importance to atmospheric physics and climate.

Open questions in the field of atmospheric aerosol microphysics include: To what degree do fractal soot aggregates collapse in response to partitioning of semi-volatiles with varying surface tensions? On what timescales do various BrC chromophores form and age? How do these timescales depend on environmental conditions? What is the role of the particle surface in the BrC chemistry for reactions at liquid or solid particle surfaces? Addressing these questions requires improved measurement technology for discerning the kinetic timescales of changes to aerosol light scattering and absorption, and the synergy of field observations with systematic laboratory studies. While progress in such technologies has made strides in recent years, widening capability and applying to kinetic studies of BrC chemistry remain amongst the immediate frontiers.

## Aerosol chemistry and microphysics drives changes in microorganism survival in aerosols

An accurate representation of aerosol microphysics is increasingly considered to be crucial to understand the role of bioaerosol as, for example, ice nuclei or in the airborne transmission of respiratory pathogens. Indeed, the same physicochemical processes that influence chemical reaction rates in droplets and aerosol optical properties are thought to impact the viability and infectivity of microorganisms in airborne particles^12^. Understanding the underlying physical chemistry of aerosols is recognised as a significant area of uncertainty in quantifying risks of airborne disease transmission^13^.

Droplets generated in the high humidity of the respiratory tract contain considerable moisture. When exhaled as respirable size particles (<5 μm in diameter) or larger droplets (typically up to sizes >100 μm) when someone speaks, coughs, or sneezes^14^, competition between evaporation, sedimentation, and forward momentum governs their airborne lifetime^15^. While 1 μm diameter droplets can remain suspended for many hours, sedimenting only 1 m in 8 h, larger droplets settle in seconds before they have lost their excess moisture and fall within a few metres from the source depending on the nature of the exhalation jet^16^. The rapid loss of moisture to equilibrate with the environment leads to a rapid increase in solute concentration (e.g., a rapid increase in ion concentrations in saliva droplets) and increased osmotic stress, particularly at the droplet surface^12^. The low mobility of relatively large bacteria or viruses ensures they are swept up by the rapidly receding droplet surface. In addition, the evaporation process drives a large temperature suppression due to evaporative cooling. The impact of cumulative osmotic stress (reflecting how quickly salt concentrations rise) has been suggested as the reason for the non-monotonic dependence of viral survival on RH, which is often at its lowest at intermediate RH values; bacteria, by contrast, seem to show a steady decline in survival as the RH decreases from wet to dry conditions^17^.

Once the aerosol containing the microorganism is airborne, pathogens continue to be influenced by their environment^12^. Outdoor air is recognised to be more toxic to aerosolised bacteria and viruses than indoor air, and this is often attributed to the “open air factor”, a term which may describe the collective impact of atmospheric oxidants such as ozone and other pollutants. Light–aerosol interactions are also critical in determining the germicidal action of ultraviolet light on the viability of airborne pathogens, although quantitative studies are lacking.

Open questions in this area include: How do droplets of respiratory fluids (e.g., saliva, deep lung fluid, or surrogates) behave during generation and evaporation, and how does their moisture content vary with time? How does the survival of microorganisms depend on droplet size, recognising that typical size ranges from respiratory events span an equivalent of 2–3 orders of magnitude in particle surface-to-volume ratio? How do environmental factors control microorganism survival from the point of exhalation and during atmospheric aging?

## Outlook

Aerosols are increasingly recognised as important sources of uncertainty in our understanding of multiple aspects of our world, whether it is atmospheric composition, disease transmission, or chemical reactivity. Although aerosol science has been dominated traditionally by atmospheric scientists and engineers, over recent decades efforts in aerosol science have blossomed to a much broader group of disciplines. Over the next 5–10 years, we expect a growing need for precise understandings of aerosol microphysical properties for applications beyond those most closely associated with aerosol science. Along with these new areas, refined approaches to characterise aerosol physical properties will undoubtedly be developed, benefitting research across a broad range of disciplines.
